# Clinical Audit to Evaluate the Quality of GP Letters From Psychiatry Clinic at TEWV NHS Foundation Trust

**DOI:** 10.1192/bjo.2025.10553

**Published:** 2025-06-20

**Authors:** Roseline Bennet Ataria, Samuel Fayomi, Harsyini Sugumaran, Clare Morgans, Susan Podmore

**Affiliations:** 1 Tees Esk and Wear Valley NHS Trust, Darlington, United Kingdom; 2 County Durham and Darlington NHS FoundationTrust, Darlington, United Kingdom

## Abstract

**Aims:** This audit aims to evaluate the quality of review letters from Psychiatry clinics against standardized practices in clinical communication with General Practices (GPs). The evaluation is based on the guidelines issued by the Royal College of Psychiatrists regarding GP letters in January 2021.

The 10 main domains letters will be judged by are:

1 – Contact details for the psychiatrist.

2 – Venue and date of meeting.

3 – Attending people + care coordinator/Lead professional.

4 – Diagnosis of the patient.

5 – Medications and allergies.

6 – A record of difference of opinion between the patient and the psychiatrist “the consultation”.

7 – Mental state examination and risk.

8 – Agreed on Plan that includes (changes in medication + treatment + follow up plan).

9 – User friendly evidence-based information and relevant links.

10 – Copying the patient into the letter/or asked if patient would like a copy.

**Methods:** The audit was conducted from December 2023 to January 2024. A Medical Secretary compiled 30 clinic letters sent to GP practices over three months, and auditors assessed compliance with Royal College guidelines using specified audit tool parameters. All audit data was collected from service users’ letters on the hospital system between September and December 2023.

**Results:** Good Practice:

100% (green compliance): Psychiatrist contact details, review date, consultation, participants, and agreed plan documented.

97% (green compliance): Venue and diagnosis documented.

93% (green compliance): Medications documented.

Issues Identified:

60% (amber compliance): Care coordinator details included.

70% (amber compliance): MSE included.

50% (red compliance): Risk assessment included.

13% (red compliance): Allergies documented.

23% (red compliance): User-friendly info/links included.

3% (red compliance): Service users copied in.

**Conclusion:** Based on the audit findings and identified issues, the following actions will be implemented:

Update GP Letter Template: Add sections for Allergies, Risk, User-Friendly Information, and Copying Patients into Letters, adhering to the Royal College of Psychiatrists’ January 2021 guidelines.

Standardize Letters: Ensure all prescribers use the updated template to maintain consistency.

Dissemination Plan: Present the updated template during team huddles.

Provide it to new trainees during handovers.

Laminate and place templates in all prescribers’ offices for reference.

Distribute the template via email to the team.

